# Comfort

**Published:** 1869-08

**Authors:** 


### COMFORT.

THE great end and aim of the mass of mankind is, to get money enough ahead to make them "comfortable;" and yet, a moment's reflection will convince us that money can never purchase "comfort"—only the means of it. A man may be "comfortable" without a dollar; but to be so, he must have the right disposition, that is, a heart and a mind in the right place. There are some persons who are lively, and cheerful, and good-natured, kind and forbearing in a state of poverty, which leans upon the toil of to-day for to-night's supper, and the morning's breakfast. Such a disposition would exhibit the same loving qualities in a palace, or on a throne.

Every day we meet with persons, who in their families are cross, ill-natured, dissatisfied, finding fault with everybody and everything, whose first greeting in the breakfast-room is a complaint, whose conversation seldom fails to end in an enumeration of difficulties and hardships, whose last word at night is an angry growl. If you can get such persons to reason on the subject, they will acknowledge that there is some "want" at the bottom of it; the "want" of a better house, a finer dress, a more handsome equipage, a more dutiful child, a more provident husband, a more cleanly, or systematic, or domestic wife. At one time it is a "wretched cook," which stands between them and the sun; or a lazy house-servant, or an impertinent carriage-driver. The "want" of more money than Providence has thought proper to bestow, will be found to embrace all these things. Such persons may feel assured that, *People who cannot make themselves really comfortable in any one set of ordinary circumstances, would not be so under any other.* A man who has a canker eating out his heart, will carry it with him wherever he goes; and if it be a spiritual canker, whether of envy, habitual discontent, unbridled ill-nature, it would go with the gold, and rust out all its brightness. Whatever a man is to-day with a last dollar, he will be radically, essentially, to-morrow with millions, unless the heart is changed. Stop, reader, that is not the whole truth, for the whole truth has something of the terrible in it. Whatever of an undesirable disposition a man has to-day without money, he will have to-morrow to an exaggerated extent, unless the heart be changed: the miser will become more miserly; the drunkard, more drunken; the debauchee, more debauched; the fretful, still more complaining. Hence, the striking wisdom of the Scripture injunction, that all our ambitions should begin with this: "Seek *first* the kingdom of God and his righteousness;" that is to say, if you are not comfortable, not happy *now*, under the circumstances which surround you, and wish to be more comfortable, more happy, your first step should be to seek a change of heart, of disposition, and *then* the other things will follow—WITHOUT THE GREATER WEALTH! And having the moral comfort, bodily comfort, bodily health will follow apace, to the extent of your using rational means. Bodily comfort, or health, and mental comfort have on one another the most powerful reactions; neither can be perfect without the other, at least, approximates to it; in short—CULTIVATE HEALTH AND A GOOD HEART; for with these you may be "comfortable" without a farthing: without them, never!—although you may possess millions!

